# Analysis of Expressed Genes of the Bacterium ‘*Candidatus* Phytoplasma Mali’ Highlights Key Features of Virulence and Metabolism

**DOI:** 10.1371/journal.pone.0094391

**Published:** 2014-04-11

**Authors:** Christin Siewert, Toni Luge, Bojan Duduk, Erich Seemüller, Carmen Büttner, Sascha Sauer, Michael Kube

**Affiliations:** 1 Division Phytomedicine, Department of Crop and Animal Sciences, Humboldt-Universität zu Berlin, Berlin, Germany; 2 Max Planck Institute for Molecular Genetics, Berlin, Germany; 3 Institute of Pesticides and Environmental Protection, Belgrade, Serbia; 4 Julius Kuehn Institute, Federal Research Centre for Cultivated Plants, Institute for Plant Protection in Fruit Crops and Viticulture, Dossenheim, Germany; Agriculture and Agri-Food Canada, Canada

## Abstract

‘*Candidatus* Phytoplasma mali’ is a phytopathogenic bacterium of the family *Acholeplasmataceae* assigned to the class *Mollicutes*. This causative agent of the apple proliferation colonizes in *Malus domestica* the sieve tubes of the plant phloem resulting in a range of symptoms such as witches’- broom formation, reduced vigor and affecting size and quality of the crop. The disease is responsible for strong economical losses in Europe. Although the genome sequence of the pathogen is available, there is only limited information on expression of selected genes and metabolic key features that have not been examined on the transcriptomic or proteomic level so far. This situation is similar to many other phytoplasmas. In the work presented here, RNA-Seq and mass spectrometry shotgun techniques were applied on tissue samples from *Nicotiana occidentalis* infected by ‘*Ca*. P. mali’ strain AT providing insights into transcriptome and proteome of the pathogen. Data analysis highlights expression of 208 genes including 14 proteins located in the terminal inverted repeats of the linear chromosome. Beside a high portion of house keeping genes, the recently discussed chaperone GroES/GroEL is expressed. Furthermore, gene expression involved in formation of a type IVB and of the *Sec*-dependent secretion system was identified as well as the highly expressed putative pathogenicity–related SAP11-like effector protein. Metabolism of phytoplasmas depends on the uptake of spermidine/putescine, amino acids, co-factors, carbohydrates and in particular malate/citrate. The expression of these transporters was confirmed and the analysis of the carbohydrate cycle supports the suggested alternative energy-providing pathway for phytoplasmas releasing acetate and providing ATP. The phylogenetic analyses of malate dehydrogenase and acetate kinase in phytoplasmas show a closer relatedness to the *Firmicutes* in comparison to *Mycoplasma* species indicating an early divergence of the *Acholeplasmataceae* from the *Mollicutes*.

## Introduction

Phytoplasmas were assigned to the provisional genus ‘*Candidatus* Phytoplasma’ belonging to the family *Acholeplasmataceae* in the class *Mollicutes* [1]. They are characterized as wall-less phytopathogenic bacteria colonizing the plant phloem and depending for spread on phloem-sucking insects in general. Phytoplasmas are associated to diseases of more than 1000 plant species [2] including many important crops such as rice (‘*Ca*. P. oryzae’), sugarcane (‘*Ca.* P. graminis’), corn (‘*Ca.* P. solani’), grapevine (e.g. ‘*Ca*. P. solani’, ‘*Ca*. P. australiense’), lime (‘*Ca*. P. aurantifolia’), grapevine (‘*Ca*. P. vitis’), deciduous fruit trees such as stone fruits (‘*Ca*. P. prunorum’), pear (‘*Ca*. P. pyri’) and apple (‘*Ca*. P. mali’) [3]–[10]. ‘*Ca.* P. mali’ is the causative agent of apple proliferation in *Malus domestica* occurring in most European countries [9]. Infections cause a reduced fruit weight (40–70% of the regular weight), unsatisfactory colouring and poor taste resulting in up to 80% unmarketable fruits [11]. Losses caused by this pathogen and closely-related phytoplasmas were estimated by € 100 million for Italy and € 25 million for Germany in 2001 [12].

Analysis of metabolism was mainly performed by metabolic reconstruction of the genome content of completely determined phytoplasma genomes comprising ‘*Ca*. P. asteris’ strains OY- M and AY-WB [13], [14], ‘*Ca*. P. australiense’ strain rp-A [15] and ‘*Ca*. P. mali’ strain AT [16] and their comparative analysis [17]. Recently a second strain of ‘*Ca*. P. australiense’ named Strawberry lethal yellows phytoplasma (CPA) str. NZSb11 was published [18]. In addition, several genomic draft sequences were determined but do not provide data on gene expression [19]–[21]. With a few exceptions, such as the partial encoded citrate metabolism of peanut witches’-broom [20], data indicate the shared genetic metabolic core repertoire of phytoplasmas as described before [17]. The genome of ‘*Ca*. P. mali’ encodes the smallest chromosome of the completely determined phytoplasmas. Furthermore, it is characterized by its linear organization and terminal repeats. Beside these terminal structures, this condensed chromosome is, in contrast to other phytoplasmas, characterized by a comparable low number of duplication and integration events.

In contrast to the progress made in genome research, only a few studies [22] investigated the overall gene expression in phytoplasma so far. The differential expression analysis based on a microarray of ‘*Ca*. P. asteris’ strain OY-M resulted in 246 differentially expressed genes in plant host and insect vector [23]. Majority of work was focused on selected genes, for instance on gene content of complex transposons enlarging the phytoplasma genomes [24], secreted effector proteins [25], [26] and other virulence-related factors such as AAA+ ATPases and HflB/FtsH proteases [27], or genes involved in pathogen-host interaction [28]. Studies on gene expression with impact on metabolic features were analysed for the degeneration of genes involved in biosynthesis of folate [29] and a partial encoded sucrose phosphorylase (*sucP*) [21]. Studies on the phytoplasma transcriptome by sequencing (e.g. RNA-Seq) are missing and only one study examined the proteome of mulberry dwarf phytoplasma but without an available corresponding genomic data set resulting in a limited interpretation of the data [22]. Nevertheless, 209 proteins were assigned to mulberry dwarf phytoplasma proteins by similarity searches.

Here, we focus on the overall gene expression of ‘*Ca.* P. mali’ strain AT by transcriptomic and proteomic approaches applying RNA-Seq and shotgun proteomics, respectively. Data analysis in combination with the reference sequence of ‘*Ca.* P. mali’ allows for the first time validation of results obtained from bioinformatical analyses of this pathogen. Subsequent comparative and phylogenetic analysis of key genes provides new insights into virulence, carbohydrate metabolism and evolution of phytoplasmas.

## Methods

### Plant material

Plants infected by ‘*Ca*. P. mali’ strain AT were kindly provided by the Julius Kuehn Institute for Plant Protection in Fruit Crops and Viticulture, Dossenheim, Germany. This material consisted of seed-grown, graft-inoculated *Nicotiana occidentalis* plants. 400 mg midribs were excised from symptomatic leaves and used for proteome and transcriptome analyses. The donor plants showed severe symptoms such as chlorosis, small leaves, crinkling and declined three months after inoculation. In addition, symptomatic plant material from *Malus domestica* cv. Golden Delicious (leaf midribs obtained from two years old trees, graft-inoculated in the previous year; height of 1.3 m) and *Catharanthus roseus* (entire leaves) were examined. Healthy control plants from all species were included in the RT-PCR and standard PCR experiments for identification of selected genes (data not shown).

### RNA-Seq and transcriptome analysis

For the extraction of RNA, tissue was ground in meshbags (Bioreba, Reinach, Switzerland) and total RNA isolation was performed using Solution-D [30]. RNA was concentrated by isopropanol precipitation, washed in 80% ethanol and the pellet resuspended in RNase-free water. Total RNA was purified and a DNase-digest applied using the NucleoSpin RNA II kit according to the manufacturer's instructions (Machery & Nagel, Oensingen, Switzerland). RiboMinus plant kit (Invitrogen, Darmstadt, Germany) was applied according to the manufacturer's instruction to decrease the amount of host plant rRNA. RNA quality was measured on an Agilent 2100 Bioanalyzer using the RNA 6000 pico kit (both Agilent, Waldbronn, Germany). Single read sequencing was performed by Illumina's sequencing by synthesis approach (RNA sample preparation kit, single read cluster generation kit v4, TruSeq SBS kit v5). Barcoded libraries were sequenced on a Genome Analyzer IIx (Illumina, San Diego, California, U.S.A.) in a single read multiplex run using a half lane of the flow chamber.

Obtained RNA-Seq reads without ambiguous read position were mapped (minimal alignment length of 90% and identity of 80%) on the genome sequence of ‘*Ca*. P. mali’ (acc. no. CU469464) excluding the 3′-end of the terminal repeat (position 559012.601943). As this region is identical to the upstream terminal repeat of the chromosome, it is only treated when required in the following. Genes identified by the mapping approach were extracted using CLC Genomics Workbench V6 (www.clcbio.com). This software package was also used for read count and reads per kilobase of exon model per million mapped reads calculation (RPKM) [31]. RNA-Seq reads were deposited in European Nucleotide Archive (www.ebi.ac.uk/ena/; acc. no. PRJEB5432).

### RT-PCR of selected genes

Total RNA was isolated from 2×20 mg tissue using the RNA InviTrap® Spin Plant RNA Mini Kit according to the manufacturer's instruction (STRATEC Molecular, Berlin, Germany) except for the elution volume, which was reduced to 25 µl. RNA samples derived from the same host plant were pooled and DNA residues were removed by DNase digest (RNase-free DNase I; New England Biolabs, Frankfurt/Main, Germany). Volume of treated samples was reduced by ethanol precipitation (2.5 volumes) adding 5 µl 3M sodium acetate (pH 5.2) and 1 µl Pellet Paint® (Novagen, Darmstadt, Germany). RNA pellet was collected by centrifugation after storage for 1 h at −80°C, dried in a vacuum concentrator (Thermo Scientific, Dreieich, Germany) and resuspended in 40 µl DEPC water.

Metagenomic DNA used in control experiments was isolated by the CTAB approach [32].

Oligonucleotides for the selected genes (**Table 1**) were designed in Primer3 [33] and Primer-BLAST (http://www.ncbi.nlm.nih.gov/tools/primer-blast/primerinfo.html) using the reference sequence of ‘*Ca*. P. mali’ (see above) and approved by PCR on genomic DNA isolated following the instructions of the manufacturer's (Thermo Scientific 2X PCR Master Mix; Thermo Scientific, Dreieich, Germany) (data not shown). In addition, DNase-treated total RNA templates were used for PCR to verify DNA absence (data not shown). RT-PCR was performed applying 70 ng DNase I treated total RNA and the OneStep RT-PCR Kit (Qiagen, Hildesheim, Germany) according to the manufacturer's instruction. Reverse transcriptase reaction (50°C for 30 min) was followed by denaturation at 95°C for 15 min. PCR reactions were performed in 35 cycles consisting an initial denaturation at 95°C for 1 min, annealing at 50°C for 30 s and an elongation at 72°C for 1.5 min. A single terminal extension was applied for 10 min at 72°C. Amplification products were separated by gel electrophoresis for quality and size control. Gene assignment was confirmed by sequencing (data not shown) and BLAST comparison [34] to the reference sequence.

**Table 1:** Primer pairs used in (q)RT-PCR reactions.

Gene	Oligonucleotides	Expected size
***long amplicons***		
*pgi*	5′- TGA ACC GGC TAT TGC ATT TCG -3′	183 bp
	5′- ACA CTA AAA CGT CCA CTG ACA GA -3′	
*pfkA*	5′- TCA ATA AAA CGT GAA GTC CCT AAA A -3′	187 bp
	5′- CAC CAG GAA TGA ACG CAG C -3′	
*fba*	5′- TCT TCT TCC TTA CCA ATA CCA CCA -3′	180 bp
	5′- TCA CGG CAG TTT TGA AGG AGT -3′	
*tpiA*	5′- CTT CTT GGT CTA TTG GTA CAG GAG -3′	200 bp
	5′- ACC AAC TAA AAC TCC ATC AAT TTC T 3′	
*pduL*	5′- TCA TAG CGG ATA GAC ATA TTC A -3′5′- AAA AGC ACA CGC ATC ATC TG -3′	185 bp
*ackA*	5′- ACT TCT ATG GGA TTC ACG CCA -3′	180 bp
	5′- TCT AGC ATC ATT AGA AAC ACC TGA -3′	
*degV*	5′- GGC ATC GTT GTA GAC TCT GCT -3′5′- AGG ACT CGG TTG ACT AGT TTT T -3′	183 bp
SAP11-like	5′- ATC ATG TTG AGA TGA TGA ACT TCC -3′	199 bp
	5′- GTC GGT TTC TTC AAA AAG AGA AAC A -3′	
*rplT*	5′- TTT GTA CCT GCG AGA CAT CG -3′	185 bp
	5′- TCT TTT TCT TTG TTT ACG ATC C -3′	
*glnS*	5′- GAG CGA TCC AAG AAT GCC TA -3′	193 bp
	5′- CAA AGG ATT TAT TAC CGC CAT C -3′	
***short amplicons***		
*pduL*	5′- GGT TTT GTT GCT CCG GTT AG -3′	82 bp
	5′- TCT ATT TGT CCT TTT GGC CCT A -3′	
*ackA*	5′- TGT TTG TCA TGC TGG TAA CGG CG -3′	89 bp
	5′- CCC TCG AGT GGC GTG AAT CCC -3′	
*sfcA*	5′- CCG GGA GTA GCT GAA CCT TGT CG -3′	119 bp
	5′- ACC TAA ACC TAA AAC CGC AGT CCC A -3′	

Selected genes were examined by Real-time quantitative reverse transcription PCR (qRT-PCR) using the Kit *Power* SYBR® Green RNA-to-C_T_™ 1-Step (Life technologies, Darmstadt, Germany) and the cycler StepOnePlus (Life technologies, Darmstadt, Germany) performing a relative qRT-PCR. Amplicons with a size of 180 to 200 bp were generated from the genes rplT, ATP_00189 (SAP11-like), pduL, pfkA and tpiA to perform a relative qRT-PCR, whereas rplT was used as endogenous control. Furthermore, qRT-PCR on the transcripts of the genes pduL, ackA and pgi, using pduL as endogenous control, was performed applying primer pairs resulting in shorter amplicons.

Each 25 µl reaction was composed of 0.2 µl RT enzyme Mix (125x), 12.5 µl RT-PCR Mix (2x), 1.0 µl forward and reverse primer each (10 ng/µl), 7.8 µl nuclease-free water and 2.5 µl DNA-free RNA (long amplicons qRT-PCR: each 87,0 ng/µl, short amplicons qRT-PCR: each 96,7 ng/µl) template obtained from the experimental host *Niccotiana occidentalis*, *Malus domestica* and *Catharanthus roseus* infected by ‘*Ca*. P. mali’ strain AT. The qRT-PCR run was performed at 48°C for 30 min, 95°C for 10 min followed by 40 cycles of amplification (95°C; 15 s; 50°C (long amplicons) and 55°C, 1 min (short amplicons). Each sample and water control was run in replicates. C_T_ values of each gene were normalized using *rplT* for long amplicons and *pduL* for short amplicons as endogenous control (StepOne™ Software v2.2.2; Life technologies, Darmstadt, Germany). ΔCT, used as CT (Cycle threshold) value of a sample was normalized with respect to the endogenous control, and average (Ø) CT, were calculated by the instrument's software.

### Protein extraction, measurement and assignment

Proteins from phloem-rich tissue (leave veins or stem) of three phytoplasma positive plants were extracted by homogenizing plant material in RLT-lysis buffer (Qiagen, Hildesheim, Germany) or self-made SDS-based lysis buffer with either mesh-bags and handhomogenizer (BIOREBA, Reinach, Switzerland) or the TissueLyser (Qiagen, Hildesheim, Germany). Prior to standard SDS-PAGE, the proteins in RLT-lysis buffer were purified with the AllPrep DNA/RNA/Protein Mini Kit (Qiagen, Hildesheim, Germany), whereas the other lysates were used directly. Disulfide bridges were reduced and alkylated either prior to SDS-PAGE or afterwards in-gel with dithiothreitol and iodacedamid. After Coomassie staining of the gel, bands were excised and cut from high to low molecular weight into 16 equally sized slices. Each slice was further dissected into small pieces of no larger than 1 mm^3^ and destained. The in-gel tryptic digest was performed as described by Shevchenko *et al.* [35] with minor modifications. Peptides were extracted from the gel using acetonitrile, vacuum dried and resuspended prior to LC-MS/MS in 5% acetonitrile, 2% formic acid. LC-MS/MS was performed via nanoflow reverse phase liquid chromatography (RPLC) (Agilent Technologies, Böblingen, Germany) in line with a Linear Ion Trap (LTQ)-Orbitrap XL mass spectrometer (Thermo Scientific, Schwerte, Germany) as described elsewhere [36], [37].

Raw files from MS analysis were processed and analyzed using the MaxQuant computational proteomics platform version 1.1.1.25 [38] which makes use of the search engine Andromeda [39]. Three missed cleavages were allowed besides otherwise standard settings including a peptide and protein false discovery rate of 1%. MS/MS spectra were searched against a target decoy database containing plant proteins and phytoplasma proteins (9,002 protein sequences of the genus *Nicotiana* extracted from NRPROT database, www.ncbi.nlm.nih.gov; 497 for ‘*Ca.* P. mali’ strain AT, acc. no. CU469464). Additionally, 13,080 protein sequences were inferred via six frame translation of all open reading frames from the genomic sequence of ‘*Ca.* P. mali’ strain AT. This data set was also included. Only protein groups identified with at least two peptides, one of them unique, and a minimum length of six amino acids, were considered. Proteomic data assigned to ‘*Ca*. P. mali’ was deposited in the PeptideAtlas database (http://www.peptideatlas.org/; acc. no. PASS00377).

### Functional analyses of expressed genes

Deduced peptide sequences of expressed genes were assigned to COG categories [40]. Selected proteins were also compared against InterPro database [41] implemented in Blast2GO [42] and Pfam including PfamB [43]. Phobius was used for prediction of signal peptides and transmembrane regions [44].

### Data derived from the mulberry dwarf phytoplasma proteome

Mulberry dwarf phytoplasma proteins previously assigned to *Tenericutes* database entries [22] were included in this study by BLASTP analysis using the annotated proteins of ‘*Ca*. P. mali’ as database [34]. MSPcrunch was used for removal of hits exceeding the probability treshhold of 1e-5 and showing a sequence identity below 25% [45].

### Proteins specific to Acholeplasmataceae

Proteins only identified in the genus ‘*Ca*. Phytoplasma’ were determined by BLASTP comparison of the deduced proteins of ‘*Ca*. P. mali’ (acc. no. CU469464) against a modified NRPROT database (www.ncbi.nlm.nih.gov, database release 2013/07/09) excluding all entries assigned to the order *Acholeplasmataceae*. Subsequently, the proteins were analysed using Megan [46] to obtain taxonomical assignment of the best hit applying modified LCA parameters (support 1, low complexity filter off). Information on expressed genes were visualised in Artemis [47].

### Phylogenetic analysis of selected genes

Malate dehydrogenase (SfcA) and acetate kinase A (AckA) were used for phylogenetic analysis. The peptide sequences were aligned using Clustal W [48] from the Molecular Evolutionary Genetics Analysis program MEGA6 [49].

The evolutionary history was inferred based on malate dehydrogenase deduced peptide sequences of available phytoplasmas and representatives of the closest species in which malate dehydrogenase is encoded *Clostridium botulinum* (strains ATCC3502 and ATCC19397), *Bacillus subtilis* subsp. *subtilis*, and *Bacillus cereus* using the Maximum Parsimony (MP) method (MEGA6). The MP tree was obtained using the Close-Neighbor-Interchange algorithm with search level 5, in which the initial trees were obtained with the random addition of sequences (ten replicates). The “Gaps/Missing Data Treatment” option was set to “use all sites”. To estimate the statistical significance of the inferred clades, 1,000 bootstrapping was performed to estimate the stability and support for the inferred clades. *Escherichia coli* strain K12 was designated as outgroup to root the tree.

In analyses of acetate kinase deduced peptide sequences of available phytoplasmas, *Mollicutes* available in HAMAP database, *Spiroplasma citri*, and representatives of *Firmicutes*: *Clostridium botulinum*, *Erysipelothrix rhusiopathiae*, *Bacillus subtilis* subsp. *subtilis*, *Lactobacillus plantarum*, *Enterococcus faecalis*, *Streptococcus pneumonia*, and *Lactococcus lactis* subsp. *lactis* were included, employing *Escherichia coli* and *Salmonella typhimurium* as outgroup. Peptide sequences of AckA were inspected in HAMAP-Scan [50] and proteins were selected assigned to encode a trusted acetate kinase profile excluding proteins encoding the propionate kinases motif, which show a high sequence identity to AckA proteins but differ in function. The evolutionary history was inferred using MP and maximum likelihood (ML) methods (MEGA6). The MP tree was obtained as described for malate dehydrogenase, while for ML tree a sequence evolution model was first chosen using the “find best model” option in MEGA6. Initial tree(s) for the heuristic search were obtained automatically. For both analyses the “Gaps/Missing Data Treatment” option was set to “use all sites”. To estimate the statistical significance of the inferred clades, 1,000 bootstrapping was performed to ensure the stability and support for the inferred clades.

## Results and Discussion

### Gene expression identified by transcriptomic and proteomic approaches

The RNA-Seq approach resulted in 17,046,418 reads with an average length of 115 b. Only 468 reads (0.003%) of all reads could be mapped against the protein coding genes of ‘*Ca*. P. mali’. The majority of reads is assigned to the plant background (analysis in progress). Of the 497 proteins annotated in the ‘*Ca*. P. mali’ genome [16], 132 were identified to be transcribed (**Information S1**). They include sixteen genes located in the two terminal repeat regions. Nevertheless, these relatively few genes represent the highest number of phytoplasma transcripts identified in one experiment so far. Except for the *rRNA* genes, the highest number of assigned RNA-Seq reads for a gene was that of a conserved hypothetical protein representing a predicted integral membrane protein ATP_00169 (42 assigned reads). However, no assumption is made on expression levels in detail derived from RNA-Seq due to the low read coverage of phytoplasmas genes and absent biological and/or technical replications. Assigned genes should be considered as highly expressed as described in other work [51].

The proteomic approach allowed the identification of 104 proteins (21%), including 6 proteins localized in the terminal repeats. In summary, expression of a non-redundant set of 208 genes was confirmed on transcriptome or proteome level. Expression of 28 genes was confirmed by both approaches.

**Figure 1:**
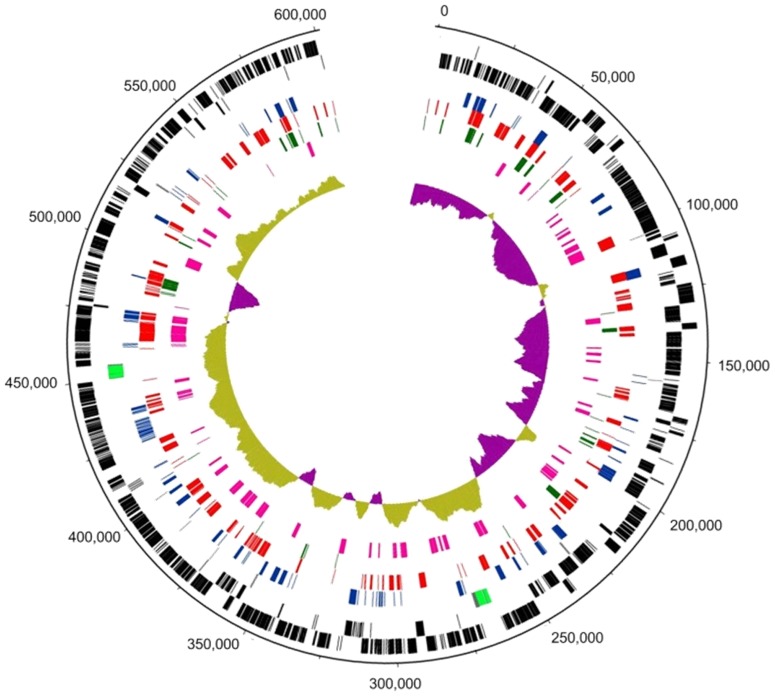
Genome circle of ‘*Ca*. P. mali’ strain AT highlights gene expression. Circular patterns (from outside to inside): 1 (outer circle), scale in base pairs of the chromosome; 2 (black), predicted protein coding sequences; 3, tRNAs (grey) & rRNAs (green); 4 (dark blue), identified proteins of ‘*Ca*. P. mali’; 5 (red), expressed genes of ‘*Ca*. P. mali’ identified by RNA-Seq; 6 (dark green), expressed genes (proteome and transcriptome) without similarity to NRPROT entries excluding the *Acholeplasmataceae* entries; 7 (magenta), assigned proteins of the mulberry dwarf phytoplasma; and 8 (olive and pink), G + C skew. Expressed genes located in the terminal ends (identical in sequence) were marked twice.

The chromosome of ‘*Ca*. P. asteris’ strain OY-M encodes 752 protein coding genes (acc. no. AP006628.2). Oshima and colleagues [23] had shown that 246 genes of this data set show a differential expression profile in plant and insect. About 63% of these genes (156 genes) are also encoded in ‘*Ca*. P. mali’. Of the 209 proteins identified in the mulberry dwarf phytoplasma proteome [22], 67 could be assigned on the genome of ‘*Ca*. P. mali’. On the other hand, 33 proteins identified in ‘*Ca*. P. mali’ are also present in the mulberry dwarf phytoplasma proteome (**Figure 1**).

### Assignment of the deduced proteins to functional groups

**Figure 2:**
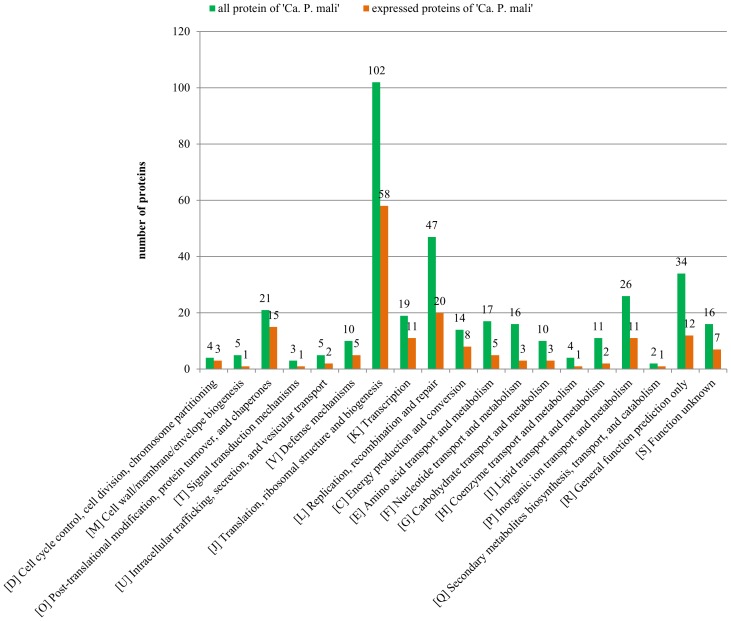
COG categories assigned to expressed gene products identified by RNA-Seq and/or proteome analysis (orange) versus the deduced protein content of '*Ca*. P. mali' strain AT (green). Values indicate total number reached in each category.

Transcripts derived from 93 genes (including 5 genes located in the terminal repeats) and 86 identified proteins (including 4 genes located in the perfect inverted terminal repeats) could be assigned to a functional Cluster of Orthologous Groups (COGs) category (**Figure 2**). Highest numbers of assignment were in the categories (I) translation, (II) replication, recombination and repair, (III) posttranslational modifications and (IV) transcription.

Highest number was obtained for the ribosomal proteins of which 42 out of 52 non-redundant proteins were identified, 10 by the transcriptome and 38 by the proteome approach.

Transcripts assigned to the two *rRNA* operons of ‘*Ca.* P. mali’ comprising 155 RNA-Seq reads for the 16S *rRNA* gene and 639 for the 23S *rRNA* gene. However, it should be considered that read numbers might be influenced by the performed depletion of plant-derived rRNA.

### Expressed genes specific to the Acholeplasmataceae

Expressed genes were screened for further coding outside the taxon *Acholeplasmataceae*. Thirty-six genes (including 4 genes located in the perfect inverted terminal repeats) by the transcriptomic and 16 (including 2 genes located in the perfect inverted terminal repeats) by the proteomic approach could be identified by BLASTP analysis only inside the *Acholeplasmataceae* data. Thirty-two and 14 of these expressed genes do not have an assigned function.

Expressed genes comprise products with assigned function such as the abundant immunodominant protein (ATP_00050) interacting with the plant actin [28], a Zn-dependent carboxypeptidase (ATP_00016, ATP_00484), a nitroreductase-like protein (ATP_00287) and the putative telomere resolvase (ATP_00103). The latter one was suggested to be involved in replication of the linear chromosome [16].

### Location of the expressed proteins

The deduced protein sequences of 40 identified transcribed genes were predicted to contain a transmembrane region but no signal peptide (**Information S1**; **Table 2**), while one hypothetical protein carries one transmembrane region and a signal peptide indicating localization on the membrane surface. The *Sec*-dependent pathway of phytoplasmas may release 5 proteins of unknown function.

**Table 2:** Total number of proteins and expressed proteins carrying transmembrane helices (TM), TM and a signal peptide (SP) or SP.

	TM	TM + SP	SP
**‘** ***Ca*** **. P. mali’ proteins (acc. no. CU469464)**	145 (+13)	5 (+1)	29 (+4)
**Expressed proteins**	56 (+5)	1	10 (+1)
*limited to*			
**transcriptome approach**	41 (+3)	1	6
**proteome approach**	21 (+3)	0	6 (+1)

Number of genes encoding transcription and translation products located in the terminal repeats is given in brackets.

Seventy-seven of the proteins identified with the proteomic approach could be predicted to have no transmembrane region and no signal peptide indicating that these proteins remain in the cytosol of the phytoplasma cell. Twenty-one proteins were predicted to be membrane-bound carrying at least one transmembrane region but no signal peptide. Beside the immunodominant protein (Imp; ATP_00050), these proteins comprise for instance ABC transporter-like subunits such as PhnL (ATP_00013/ATP_00485), dipeptide/oligopeptide transport system component DppA (ATP_00068), subunit MetQ of the methionine transport system (ATP_00192), type IVB secretion system IcmE protein (ATP_00087), hemolysin-like protein (ATP_00276) and the Zn-dependent protease TldD (ATP_00323).

In addition, six expressed proteins identified by the proteomic approach are predicted to be secreted via the *Sec*-dependent secretion system. Five of them encode proteins without assigned function, one encodes the adenylate kinase (Adk). The prediction for Adk should be considered to be incorrect.

### Pathogen-plant host interaction

The *Sec*-dependent secretion pathway and its importance for the phytoplasma membrane proteins and protein release were discussed in the past [52]. The expression of predominant membrane proteins interacting with the host, such as Imp [28], was also confirmed by this study. In addition, the expression of the signal recognition particle protein (Ffh) and preprotein translocase subunit SecY was verified. The gene encoding the ATPase subunit SecA is also expressed as previously described for phytoplasmas [22]. The possibility of an additional type IVB secretion system in phytoplasmas was suggested after the identification of the core protein IcmE [14], [15], [19], [53]. Expression of the membrane protein IcmE (ATP_00087) was confirmed by transcript and protein analysis for the first time indicating an additional functional active secretion system that might be also involved in virulence of ‘*Ca.* P. mali’ and probably other phytoplasmas.

Of particular interest is that the expressed gene ATP_00189 encodes a predicted secreted protein similar to the pathogenicity-related effector protein SAP11 of ‘*Ca*. P. asteris’ strain AY-WB. SAP11 and the SAP11-like protein of ‘*Ca*. P. mali’ share the signal peptide motif of the phytoplasma-specific sequence-variable mosaic (SVM) protein signal sequence (Pfam entry: PF12113). SVM-carrying proteins undergo a rapid evolution [54]. In the ‘*Ca.* P. mali’ strain AT genome, the SVM motif is restricted to ATP_00189, whereas in other phytoplasma genomes several proteins show this signature in the Pfam database [43].

The proteins carrying the SVM motif are encoded in all five completely determined phytoplasma genomes although its conservation is limited (41% identity). SAP11 is one of the first identified effector proteins located in a complex transposon region (called potential mobile unit; PMU) of ‘*Ca*. P. asteris’ strain AY-WB [26]. ATP_00189 is not assigned to a PMU-like region of ‘*Ca.* P. mali’ [16]. The SAP11 protein of strain AY-WB accumulates in the plant host nuclei resulting in a change of the transcription profile. Results are a manipulation of the plant development and changes in the hormone biosynthesis [55]. Expression of SAP11 in *Arabidopsis* Col-0 lines results in crinkled leaves and siliques beside an increased number of stems. Similar symptoms occur in ‘*Ca*. P. mali’ infected *N. occidentalis* plants, *Malus domestica* trees and the phytoplasma model plant *Catharanthus roseus*.

Beside this putative effector protein, *sodA* was found to be expressed encoding an iron/manganese superoxide dismutase family protein. This protein is involved in the release of H_2_O_2_, which is described as a virulence factor for *Mycoplasma pneumoniae* [56] and was also suggested to be associated to virulence of phytoplasmas [17].

Furthermore, the genome of ‘*Ca*. P. mali’ encodes six AAA+ ATPases and five HflB proteases [27]. Recently published results indicate that several members of these two groups of AAA+ proteins are associated to strain virulence. Approximately half of these proteins show a predicted extracellular orientation and may be involved in pathogen–host interactions resulting in compromised phloem function. Expression was verified for both genes.

### Transport

Expressed genes encoding subunits of the ABC-type transporters for spermidine/putrescine transport (PotB), dipeptides (DppA) and methionine transport system (MetI, MetQ) were determined corresponding to the dependence of phytoplasmas on the uptake of external amino acids [17]. Furthermore, the ABC-type transport system for the cofactors manganese/zinc (ZnuC) and cobalt (CbiO2, CbiQ) were identified. Carbohydrate uptake is indicated by the expressed ABC-transporter for sugar (MalE) and malate by the symporter MleP.

### Expressed genes involved in metabolism

Proteome and transcriptome data also support the expression of the metabolic key genes. Expression of the two DegV family proteins (ATP00094/95) was confirmed. These two genes of unknown function are assigned to the EDD-fold superfamily (Pfam CL0245). However, crystal structure of DegV from *Thermotoga maritima* (TM841) showed the presence of a bound palmitate molecule indicating a fatty acid binding ability that may play a role in the cellular functions of fatty acid transport or metabolism [57]. DegV family proteins are encoded in many phytoplasma genomes and also *Acholeplasmataceae* such as *Acholeplasma palmae* (acc. no. CCV63690), *A. laidlawii* (acc. no. ABX80709) and *A. brassicae* (acc. no. CCV66635).

**Figure 3:**
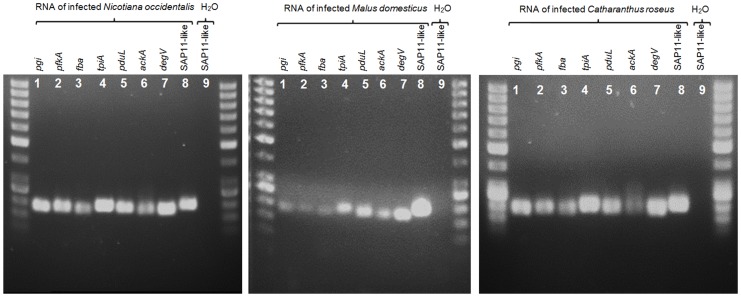
RT-PCR confirming the expression of *pgi*, *pfkA*, *fba*, *tpiA*, *pduL*, *ackA*, *degV* and SAP11-like gene. RNA was obtained from *Nicotiana occidentalis*, *Malus domestica*, *Catharanthus roseus* infected by ‘*Ca*. P. mali’ strain AT. The RT-PCR products were separated on a 1.4% TAE agarose gel. Lane number nine was used for negative control applying water as template (example SAP11-like gene). The product size of around 200 bp was estimated by the 50 bp DNA ladder (Lifetechnologies) loaded on first and last lane of each gel.

Two general pathways were suggested providing ATP to phytoplasmas [17]. They consist of the (I) Embden-Meyerhof-Parnas pathway [13] and (II) the formation of acetate [17], [58]. ‘*Ca*. P. mali’ is lacking the ATP providing part of the glycolysis [16] while the genetic repertoire for the formation of glyceralaldehyde-3-phosphate and dihydroxyacetone-phosphate is encoded. Data derived from RNA-Seq and shotgun proteomics do not provide evidence on expression of the upper part of glycolysis in ‘*Ca*. P. mali’. Expression could not be confirmed for phosphoglucose isomerase (Pgi), phosphofructo-kinase (PfkA) and the fructose-bisphosphate aldolase (Fba) by RNA-Seq and shotgun proteomics. Fba and the triphosphate-isomerase (TpiA) were identified in the proteome of the mulberry dwarf phytoplasma [22]. Transcripts of *tpiA* and of the suggested candidate for a hexose-6-phosphatase (ATP_00245) providing glucose-6-phosphate were identified in ‘*Ca*. P. mali’. Additional RT-PCR experiments applying gene-specific oligonucleotides for the amplification of *pgi*, *pfkA*, *fba*, *tpiA*, *pduL*, *ackA*, *degV* and the SAP11-like gene (ATP_00189) of ‘*Ca*. P. mali’ in *N. occidentalis*, *C. roseus* and *M. domestica* indicate expression of these genes including the upper part of the glycolysis encoded by ‘*Ca*. P. mali’ in different plant hosts (**Figure 3**).

In contrast, RNA-Seq and mass spectrometry shotgun data provide evidence for the genes involved in the suggested alternative ATP-providing pathway in phytoplasmas starting by the uptake of malate and the production of acetate [17]. In this pathway, malate is taken up by the symporter MleP (synonym CitS), undergoes oxidative decarboxylation, is converted to pyruvate by the malate dehydrogenase (SfcA) and acetyl-CoA is formed by the pyruvate dehydrogenase complex (AcoA, AcoB, AceF and LpD). An alternative entry substrate for the malate dehydrogenase (SfcA) might be oxaloacetate [16]. The PduL-like phospotransacetylase (ATP_00224) forms acetyl-phosphate and ATP is released during the acetate formation mediated by the acetate kinase (AckA). Expression of all these genes is confirmed by the proteomic approach of this study. The expression of *acoB* and the *pduL*-like gene is additionally confirmed by RNA-Seq and of *mleP* limited to RNA-Seq. These data support the importance of the suggested alternative pathway to gain ATP. Key enzymes are the malate dehydrogenase and acetate kinase. Expression levels of selected genes of ‘*Ca*. P. mali’ strain AT were examined in *Nicotiana occidentalis, Malus domestica and Catharanthus roseus*, which is used as a model plant in phytoplasma research. Relative qRT-PCR using the house keeping gene encoding the 50S ribosomal protein L20 (*rplT*) as internal control verified the high expression of ATP_00189 encoding the SAP11-like protein (**Table 3**) but also the phosphotransacetylase encoded by the PduL-like protein always reached a higher expression compared to the endogenous standard.

**Table 3:** Experimental hosts and genes used in qRT-PCR experiments with their correlating average (Ø) C_T_ and ΔC_T_ values after normalization (long amplicons).

host	*gene*	Ø C_T_	ΔC_T_
*N. occidentalis*	*rplT*	24.83	0.000
	SAP11-like	20.89	3.940
	*pduL*	23.36	1.470
	*pfkA*	22.93	1.900
	*pgi*	25.76	−0.930
*M. domestica*	*rplT*	29.43	0.000
	SAP11-like	22.97	6.465
	*pduL*	28.94	0.490
	*pfkA*	29.10	0.330
	*pgi*	31.34	−1.905
*C. roseus*	*rplT*	22.77	0.000
	SAP11-like	19.82	2.950
	*pduL*	22.74	0.025
	*pfkA*	24.64	−1.875
	*pgi*	24.96	−2.190

**Figure 4:**
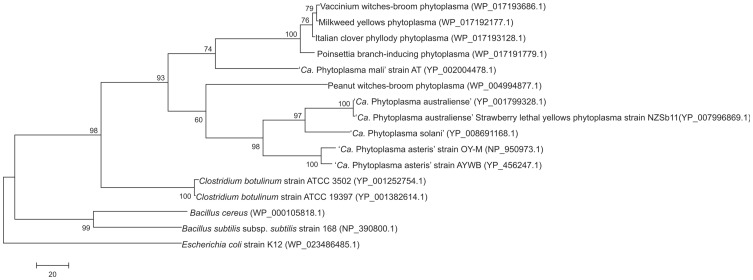
Phylogenetic tree constructed by parsimony analyses of deduced malate dehydrogenase peptide sequences of available phytoplasmas, *Clostridium botulinum* strains ATCC3502 and ATCC19397, *Bacillus subtilis* subsp. *subtilis*, and *Bacillus cereus* employing *Escherichia coli* strain K12 as outgroup. Accession numbers are given in parentheses. Numbers on the branches are bootstrap values obtained for 1,000 replicates (only values above 60% are shown). The tree is drawn to scale, with branch lengths calculated using the average pathway method, and are in the units of the number of changes over the whole sequence. The scale bar represents 20 amino acid substitutions.

The pathway depends on the availability of pyruvate, which may depend or might be provided by SfcA. Relative qRT-PCR, producing short amplicons and applying *pduL* as endogenous control, also turns out the high expression of *sfcA* in comparison to *ackA* (**Table 4**). The malate dehydrogenase was not identified in the three acholeplasma genomes or in other *Mollicutes* [58] but was identified in complete genomes and several phytoplasma draft sequences (**Figure 4**). It is suggested that pyruvate is provided in phytoplasmas by the conversion of malate catalysed by a malate dehydrogenase (SfcA) of the subgroup 2 in the presence of Mg^2+^ or Mn^2+^ and the concomitant reduction of the cofactor NAD^+^ or NADP^+^.

**Table 4:** Experimental hosts and genes used in qRT-PCR experiments with their correlating average (Ø) C_T_ and ΔC_T_ values after normalization (short amplicons).

host	*gene*	Ø C_T_	ΔC_T_
*N. occidentalis*	*pdul*	22.76	0.000
	*sfcA*	17.27	5.490
	*ackA*	24.92	−2.165
*M. domestica*	*pdul*	23.99	0.000
	*sfcA*	22.96	1.031
	*ackA*	29.91	−5.914
*C. roseus*	*pdul*	19.95	0.000
	*sfcA*	17.09	2.863
	*ackA*	26.73	−6.775

### Phylogenetic analyses of acetate kinase and malate dehydrogenase

**Figure 5:**
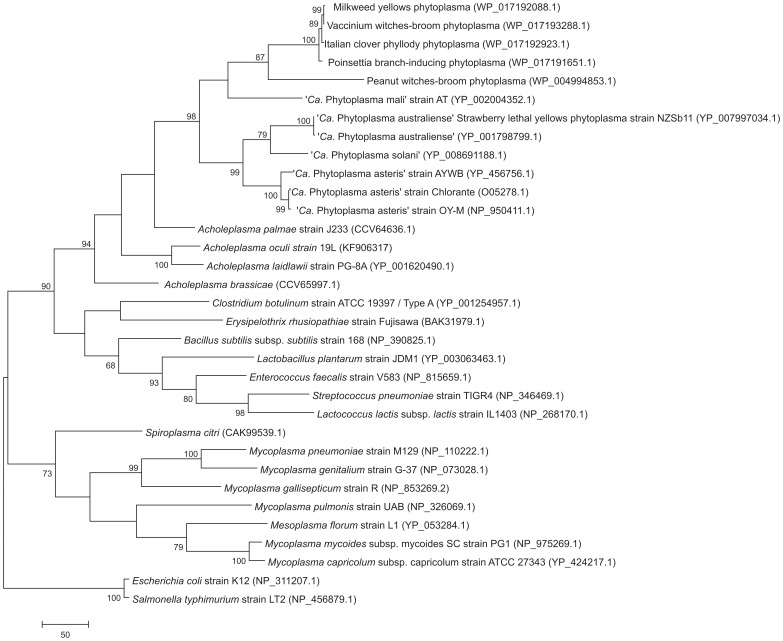
Phylogenetic tree constructed by parsimony analyses of the deduced peptide sequences of acetate kinase of available *Mollicutes* in HAMAP database, *Spiroplasma citri*, *Clostridium botulinum*, *Erysipelothrix rhusiopathiae*, *Bacillus subtilis* subsp. *subtilis*, *Lactobacillus plantarum* **,** *Enterococcus faecalis* **,** *Streptococcus pneumonia* **,** **and** *Lactococcus lactis* **subsp.** *lactis* **employing** *Escherichia coli* **and** *Salmonella typhimurium* **as outgroup.** One of the two most parsimonious trees is shown. Accession numbers are given in parentheses. Numbers on the branches are bootstrap values obtained for 1,000 replicates (only values above 60% are shown). The tree is drawn to scale, with branch lengths calculated using the average pathway method, and are in the units of the number of changes over the whole sequence. The scale bar represents 50 amino acid substitutions.

Acetate kinase (AckA) is encoded in many *Mollicutes* including the acholeplasmas [58] and current phylogenetic analysis of this protein indicates highly supported monophyly of the *Acholeplasmataceae* with topology of the clade highly congruent with its 16S rDNA phylogeny [17]. This data analysis also indicates a closer relatedness of *Acholeplasmataceae* to *Firmicutes*, in particular *Clostridium* compared to *Bacillus*, than to the remarkably separated mycoplasmas (**Figure 5**; **Information S2**). A similar phylogenetic assignment results from the malate dehydrogenase also taking part in this pathway, which is not encoded in acholeplasmas and mycoplasmas. Malate dehydrogenase shows a monophyletic origin in phytoplasmas (**Figure 4**) and analysis shows its closest relatedness to the genera *Clostridia* and *Bacillus* of the *Firmicutes* in this comparison.

## Conclusions

Transcription and protein expression data obtained from infected *N. occidentalis* tissue represent valuable information on the gene expression of ‘*Ca*. P. mali’ strain AT. The data provides for the first time insights into expression of metabolic and putative virulence-related key genes of ‘*Ca.* P. mali’. Glycolysis was described as the main pathway in *Mollicutes* [13]. However, the discovery of the absence of the energy-yielding part of glycolysis in ‘*Ca*. P. mali’ resulted in the *in silico* reconstruction of an alternative pathway from malate or a similar substrate to acetate to provide ATP for the cell [16], [17]. So far uncharacterized bypasses may allow entering this pathway from pyruvate as it encoded in acholeplasmas [58]. However, phytoplasma genomes differ from acholeplasmas by encoding the symporter for malate/citrate and the malate dehydrogenase. It is notable that the similar phylogenetic assignments, resulted from metabolic core proteins of phytoplasmas such as acetate kinase and malate dehydrogenase, indicate an early divergence and independent evolution from other *Mollicutes*. These genetic features separate the *Acholeplasmataceae* from other *Mollicutes*.

In addition to the metabolic features described, this study allowed narrowing the number of candidates of effector proteins to a SAP11-like protein in ‘*Ca*. P. mali’ strain AT. Furthermore, housekeeping proteins involved in replication and translation, that are predominant in the expressed proteins, included the chaperone GroES/GroEL. This chaperone was discussed to undergo degradation in phytoplasma evolution [19], [21]. However, this does not apply for ‘*Ca*. P. mali’.

A higher coverage of the transcriptome and its expression levels will be needed for full phytoplasma transcriptome coverage, reconstruction and allocation of starting points for further analysis. This option will be available in future considering the decreasing sequencing costs offering new perspectives in metatranscriptome analysis of phytoplasma colonizing hosts and vectors. First studies such as the profiling of the mouse intestinal metatranscriptome highlight this emerging research field [59].

## Supporting Information

Information S1 **Overview about expressed proteins including assignment of the RNA-Seq and proteome data (this study), genes assigned to be differentially expressed in ‘** ***Ca*** **. P. asteris’ strain OY-M** **[23]** **, and the proteome of mulberry dwarf phytoplasma** **[22]** **.** Information provided in columns: (1) locus tag (bold letters indicate location in the chromosomal terminal inverted repeats); (2) gene name; (3) product name; (4) number of assigned RNA-Seq reads; (5) RPKM value of the assigned RNA-Seq reads (reads per kilobase transcript per million reads, for length normalization); (6) protein identification by MS proteome shotgun (this study); (7) sequence coverage of the protein by assigned peptides (MS proteome shotgun); (8) PEP location (posterior error probability); (9+10) phobius prediction of transmembrane region(s) (TM) and a signal peptide (SP); (11) identified by other proteome studies dealing with proteomic of phytoplasmas, in particular ‘*Ca*. P. mali’ and (12) COG category assignment. Colours highlight identified proteins (green), transcripts (blue) and genes with assigned transcript and protein (red). No colour was assigned for the mulberry dwarf phytoplasma proteins assigned to ‘*Ca*. P. mali’.
(XLSX)

Information S2 **Maximum likelihood tree based on the Le_Gascuel_2008 model** **[60]** **of the deduced peptide sequences of acetate kinase of available** ***Mollicutes*** **in HAMAP database,** ***Spiroplasma citri*** **,** ***Clostridium botulinum*** **,** ***Erysipelothrix rhusiopathiae*** **,** ***Bacillus subtilis*** **subsp.** ***subtilis*** **,** ***Lactobacillus plantarum*** **,** ***Enterococcus faecalis*** **,** ***Streptococcus pneumonia*** **, and** ***Lactococcus lactis*** **subsp.** ***lactis*** **employing** ***Escherichia coli*** **and** ***Salmonella typhimurium*** **as outgroup.** The tree with the highest log likelihood (-14357.6146) is shown. The percentage of trees in which the associated taxa clustered together is shown next to the branches. Initial tree for the heuristic search was obtained by applying the Neighbor-Joining method to a matrix of pairwise distances estimated using a JTT model. A discrete Gamma distribution was used to model evolutionary rate differences among sites (5 categories (+G, parameter  = 1.4727)). The rate variation model allowed for some sites to be evolutionarily invariable ([+I], 9.6080% sites). The tree is drawn to scale, with branch lengths measured in the number of substitutions per site.
(PDF)
