# Supplementary material for: Analysis of Expressed Genes of the Bacterium ‘Candidatus Phytoplasma Mali’ Highlights Key Features of Virulence and Metabolism
Source: PLoS One. 2014 Apr 11;9(4):e94391. doi: 10.1371/journal.pone.0094391 (PMC3984173; doi:10.1371/journal.pone.0094391)
Supplement: Information S2 — Maximum likelihood tree based on the Le_Gascuel_2008 model [60] of the deduced peptide sequences of acetate kinase of available Mollicutes in HAMAP database, Spiroplasma citri , Clostridium botulinum , Erysipelothrix rhusiopathiae , Bacillus subtilis subsp. subtilis , Lactobacillus plantarum , Enterococcus faecalis , Streptococcus pneumonia , and Lactococcus lactis subsp. lactis employing Escherichia coli and Salmonella typhimurium as outgroup. The tree with the highest log likelihood (-14357.6146) is shown. The percentage of trees in which the associated taxa clustered together is shown next to the branches. Initial tree for the heuristic search was obtained by applying the Neighbor-Joining method to a matrix of pairwise distances estimated using a JTT model. A discrete Gamma distribution was used to model evolutionary rate differences among sites (5 categories (+G, parameter = 1.4727)). The rate variation model allowed for some sites to be evolutionarily invariable ([+I], 9.6080% sites). The tree is drawn to scale, with branch lengths measured in the number of substitutions per site. (PDF) [file pone.0094391.s002.pdf]

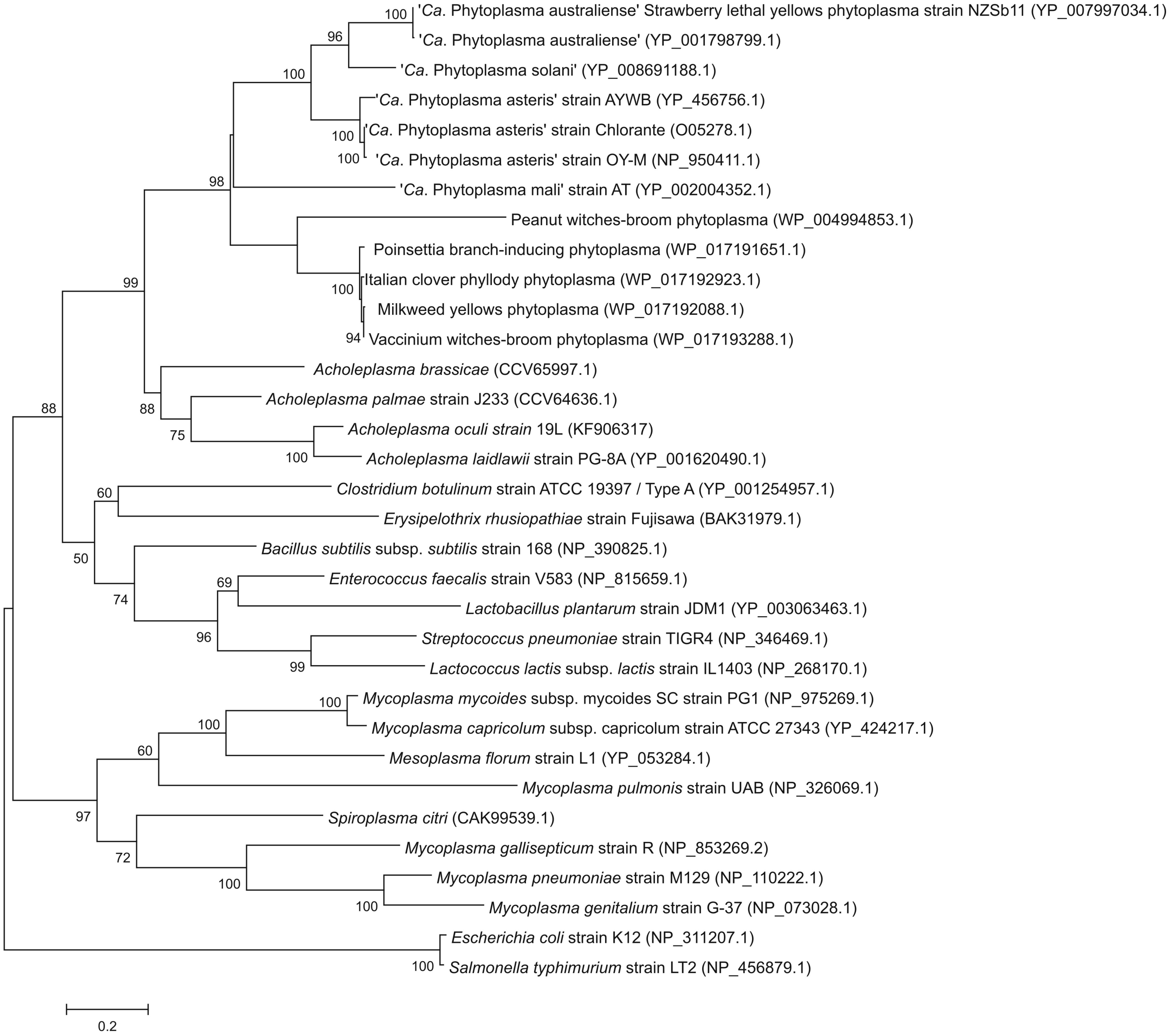

Supporting information 2. Maximum likelihood tree based on the Le_Gascuel_2008 model (Le and Gascuel, 1993) of the deduced peptide sequences of acetate kinase of available *Mollicutes* in HAMAP database, *Spiroplasma citri*, *Clostridium botulinum*, *Erysipelothrix rhusiopathiae*, *Bacillus subtilis* subsp. *subtilis*, *Lactobacillus plantarum*, *Enterococcus faecalis*, *Streptococcus pneumonia*, and *Lactococcus lactis* subsp. *lactis* employing *Escherichia coli* and *Salmonella typhimurium* as outgroup. The tree with the highest log likelihood (-14357.6146) is shown. The percentage of trees in which the associated taxa clustered together is shown next to the branches. Initial tree for the heuristic search was obtained by applying the Neighbor-Joining method to a matrix of pairwise distances estimated using a JTT model. A discrete Gamma distribution was used to model evolutionary rate differences among sites (5 categories (+G, parameter = 1.4727)). The rate variation model allowed for some sites to be evolutionarily invariable ([+I], 9.6080% sites). The tree is drawn to scale, with branch lengths measured in the number of substitutions per site.
